# Quantization-Aware NN Layers with High-throughput FPGA Implementation for Edge AI

**DOI:** 10.3390/s23104667

**Published:** 2023-05-11

**Authors:** Mara Pistellato, Filippo Bergamasco, Gianluca Bigaglia, Andrea Gasparetto, Andrea Albarelli, Marco Boschetti, Roberto Passerone

**Affiliations:** 1 Dipartimento di Scienze Ambientali, Informatica e Statistica (DAIS), Università Ca’Foscari di Venezia, Via Torino 155, 30170 Venezia, Italy; mara.pistellato@unive.it (M.P.); filippo.bergamasco@unive.it (F.B.); albarelli@unive.it (A.A.); 2 Dipartimento di Management, Università Ca’Foscari di Venezia, Cannaregio 873, 30121 Venezia, Italy; gianluca.bigaglia@unive.it (G.B.); andrea.gasparetto@unive.it (A.G.); 3 Covision Lab SCARL, Via Durst 4, 39042 Bressanone, Italy; marco.boschetti@covisionlab.com; 4 Dipartimento di Ingegneria e Scienza dell’Informazione (DISI), University of Trento, Via Sommarive 9, 38123 Trento, Italy

**Keywords:** quantized CNN, quantization-aware training, FPGA, edge AI, peak-detection

## Abstract

Over the past few years, several applications have been extensively exploiting the advantages of deep learning, in particular when using convolutional neural networks (CNNs). The intrinsic flexibility of such models makes them widely adopted in a variety of practical applications, from medical to industrial. In this latter scenario, however, using consumer Personal Computer (PC) hardware is not always suitable for the potential harsh conditions of the working environment and the strict timing that industrial applications typically have. Therefore, the design of custom FPGA (Field Programmable Gate Array) solutions for network inference is gaining massive attention from researchers and companies as well. In this paper, we propose a family of network architectures composed of three kinds of custom layers working with integer arithmetic with a customizable precision (down to just two bits). Such layers are designed to be effectively trained on classical GPUs (Graphics Processing Units) and then synthesized to FPGA hardware for real-time inference. The idea is to provide a trainable quantization layer, called *Requantizer*, acting both as a non-linear activation for neurons and a value rescaler to match the desired bit precision. This way, the training is not only *quantization-aware*, but also capable of estimating the optimal scaling coefficients to accommodate both the non-linear nature of the activations and the constraints imposed by the limited precision. In the experimental section, we test the performance of this kind of model while working both on classical PC hardware and a case-study implementation of a signal peak detection device running on a real FPGA. We employ TensorFlow Lite for training and comparison, and use Xilinx FPGAs and Vivado for synthesis and implementation. The results show an accuracy of the quantized networks close to the floating point version, without the need for representative data for calibration as in other approaches, and performance that is better than dedicated peak detection algorithms. The FPGA implementation is able to run in real time at a rate of four gigapixels per second with moderate hardware resources, while achieving a sustained efficiency of 0.5 TOPS/W (tera operations per second per watt), in line with custom integrated hardware accelerators.

## 1. Introduction

The low cost and reasonable computational capabilities offered by recent IoT (Internet of Things) devices have favored an unprecedented widespread diffusion of smart sensors, equipped with a wide range of computing power profiles. As a result, the data processing paradigm is steadily shifting from the domain of centralized computation to distributed tasks performed directly on the sensor that gathers the data. This approach is usually referred to as *edge computing*, which is a general term comprising different specific scopes. Among these, the term *edge AI* (Artificial Intelligence) has recently emerged to describe the scenario where the distributed task involves neural networks (NN) or other flavors of AI-based inference (see [1] for a comprehensive survey).

There are several advantages in performing inference on the edge rather than sending data to a central server. For starters, this enables continuous operations even when the network is not available or unreliable, which could be the case for vehicles [2,3], robots [4] or aircrafts [5]. In addition, when there are a huge number of sensors, edge AI scales much better, both in terms of communication overhead and of computation power required for centralized inference [6]. Finally, edge AI plays a central role when a fast closed loop is needed for real-time control purposes within industrial or harsh environments [7,8,9,10].

This latter scenario is exactly the context we are focusing our effort into. Specifically, we are addressing the problem of implementing convolutional neural networks (CNNs) on embedded hardware. This particular type of neural network is quite relevant with respect to edge AI; in fact, it finds applications in many different industrial settings. Among these settings, embedded and real-time computer vision is by far the most common, as the main characteristic of CNNs is their ability to relate local information in organized data structures that can be 1D (scanlines), 2D (grayscale images) or 3D (RGB (Red, Green, Blue) channels or multispectral images).

Industrial computer vision applications are also known to be quite demanding in terms of computing resources, as they usually process thousands or millions of pixels, as well as in temrs of bandwidth, as modern applications often require several camera units and high framerates. These conditions make such applications (and thus embedded CNNs) a great candidate for edge AI, since being able to process the data directly on the cameras will make the vision task scalable both in terms of computing power and data transfer [11]. Unfortunately, at the same time, the sheer size of the multidimensional data involved makes it difficult to handle the workload using the limited resources available on a typical camera controller. In fact, most traditional approaches are designed to take advantage of powerful CPU (Central Processing Unit) systems or modern GPUs (Graphics Processing Unit) [12], which makes it quite unfeasible to move the processing on the edge.

In order to overcome this quandary, two possible paths can be followed. The first involves increasing the capabilities of the embedded system. This is the case with modern GPU-based lightweight processing units, such as the Jetson, which easily allows one to port full-fledged neural networks on the edge [13,14], or downright dedicated hardware implementations of the desired algorithms [15]. The second (and opposite) path aims at reducing the size and complexity of the neural network, by taking specially crafted measures, during both the training and the inference processes.

With this paper, we follow the second route. In particular, we introduce a training and an inference method to build compact and efficient CNNs that can be adopted on very resource-limited hardware. Namely, we target Field-Programmable Gate Array (FPGA) integrated circuits. These hardware components are typically low powered, however they can be specialized to perform specific operations that we explicitly exploit in our method. Several methods, discussed below, have been proposed to run CNNs on FPGAs. We focus specifically on light-wise CNNs that can be executed directly on the camera to perform a variety of tasks in real time and meet the requirements of accuracy and high data-rate, and low-complexity. Examples are image pre-processing, peak detection, keypoint localization or descriptor computation. This results in a complete hardware component that acquires and analyzes the image, reducing communication requirements, with enhanced latency and throughput. We demonstrate the effectiveness of the resulting approach, which is based on a new integer quantization layer acting both as a non-linear activation function and a rescaler, on a computer vision task. Nevertheless, the contribution is utterly general, since nothing prevents one from applying the newly introduced framework to any CNN, regardless of the task it is applied onto.

### 1.1. Motivating Application

To motivate our work, we take the problem of *peak detection* as a case study. Peak detection is a fundamental component in many computer vision and measurement tasks, for example as a first stage in 3D laser-based triangulation systems. These 3D triangulation systems are employed in many different fields, ranging from the automotive and electronic manufacturing to the lumber mills industry. In such systems, a narrow stripe of light, emitted from a laser or LED projector, is emitted onto a 3D surface and results in an illuminated line that will appear distorted from an observing camera positioned at an angle with respect to the projector; the analysis of these line images allows the extraction of an accurate geometric reconstruction of the object shape. The need for achieving increasing accuracy and speed pushes toward the use of higher resolution cameras with high frame rates, increasing the amount of data to be processed. The current technological state of the art reaches a rate of over 5 Giga pixel/s, corresponding to a data rate of 50–60 Gbps depending on the depth and precision of the pixel representation. Moreover, it is common to have systems consisting of several cameras, in the range of 8 and up to 30 cameras, with an aggregate data rate, which is challenging in terms of data transmission and processing.

The images provided in such triangulation systems are highly redundant, since they consist typically of a homogeneous and relatively constant dark background with a low number of bright pixels, corresponding to the projected laser line. The first step of the analysis consists in finding the position of these bright lines or spots, discarding the majority of the image area, and keeping only the position and a neighborhood of a few pixels to perform a sub-pixel position refinement. This first reduction process can take place close to the sensor camera, following an edge paradigm, for example using a field programmable gate array (FPGA). A common approach consists in developing highly customized FPGA algorithms that must cope with the real-time requirements and limited hardware resources; the drawback is that the developing and tuning of such algorithms can require months of man work. A simple single- or multiple-threshold method to perform peak detection shows several drawbacks and cannot always reach good performance: the background is not uniformly dark, but it can contain regions that due to ambient illumination are brighter than the laser stripes; the reflected signals can be very weak on dark surfaces, with an extremely small pixel intensity difference; there might be reflections around the main laser line in the case of highly-reflective surfaces such as metals. However, laser stripes present a clear discriminative pattern and, although in some cases difficult to detect using simplistic classic computer vision methods, they can be effectively detected using a machine learning approach.

This paper focuses on the implementation on an FPGA architecture of a general peak detector based on convolutional neural networks (CNNs), that meets the requirements of high accuracy, high data-rate and low-complexity. This is achieved by applying quantization-aware training to reduce the hardware requirements, and by developing a dedicated streaming pipelined architecture that avoids intermediate access to memory, significantly increasing performance and efficiency. The additional benefit of the proposed approach is that the CNN hardware can be efficiently reconfigured to perform different tasks, by simply changing the weights of the network, as opposed to the effort needed for the implementation of specialized functions, a task that usually requires specific code written in complex and not widely known programming languages such as Verilog or VHDL; on the contrary, the proposed solution permits a general approach that relies on more widely known concepts based on deep learning. Because the network is quantized and pruned, the hardware requirements can generally be met with relatively low resources. On the other hand, this may pose limits in terms of the achievable accuracy and robustness of the network, compared to more traditional approaches, depending on the complexity of the application. Likewise, if a larger network is required, the streaming architecture may need to be time multiplexed on the hardware, with additional cost in terms of memory access.

### 1.2. Related Work

Implementing real-time inference on embedded devices must face the problem of executing classification and regression algorithms on resource-constrained hardware. While neural networks are generally designed using high-level frameworks, making use of accurate floating point operations, the hardware implementation follows an alternative strategy where weights and activations are *quantized* to use a fixed-point representation with fewer bits. This results in a much smaller design that can more easily fit in devices with limited resources, with reduced computation time and power consumption. In their seminal work, Jacob et al. [16] propose the idea to map the range of values of the weights and activations of each layer uniformly over integer values represented in a given number of bits. The operations are then performed on the integer values, and the output of each layer is then re-normalized for the subsequent layer. The authors describe a procedure for the quantization-aware training of the network co-designed with a quantized inference framework, which is shown to be implementable on integer-arithmetic-only hardware. Since then, several works have shown the great and consistent success of the quantization approach in both training [17,18,19] and inference [20,21,22,23,24] of neural network models.

Still, quantization is not the only means to achieve networks size reduction. In particular, one can remove from the network those units that contribute the least (also referred as *small saliency neurons*) to the final accuracy of classification, resulting in a *pruning* operation [25,26,27,28,29]. Another alternative is *compression*, which consists of indexing the space of the weights and sharing weights among different units [30]. This is especially effective to reduce the size of the memory required to store the weights. However, this only marginally affects the size of the actual computation network, with the final weights still represented (generally) as floating-point numbers.

Another technique that can be adopted to reduce the number of parameters, as well as the number of arithmetic operations, is the use of *separable convolution*. This technique consists in partitioning a two-dimensional convolution into two separate convolutions along different axes, thus reducing the parameters from $k^{2}$ to 2k, where *k* is the size of the kernel. SqueezeNext [31] is one example of an implementation using this method. This can also be applied to one-dimensional convolutions, when considering that the convolution spans both the natural input dimension and the channel depth. However, the advantages in terms of parameter reduction are much more limited. For this reason, we did not apply this method in the present exploration.

An extreme form of quantization consists in reducing the weights and the activations to a single bit [32,33,34,35]. Weights and activations are therefore essentially constrained to only have values 1 and −1, and multipliers can be replaced by XNOR gates. This extreme simplification can still produce good accuracy when the original network is sufficiently rich.

While reduction methods are a general tool to deal with neural networks’ complexity, the hardware implementation also constitutes a factor to determine the overall final performance in terms of size, latency and throughput. Several proposals have been published in terms of neural network accelerators targeting FPGA platforms or direct custom hardware implementations. Eyeriss [36] is composed of an array of custom processing elements with global buffers over which a network is mapped for execution. The results show high efficiency in terms of hardware utilization. EIE [37] implements a compressed network directly on SRAM (Static Random Access Memory), achieving considerable savings in terms of area and energy efficiency. These and other solutions are interesting and represent a reference for the design of generalized neural computation acceleration. We are particularly interested in FPGA implementations, because they can be easily integrated, have a lower access barrier and are often already present in many industrial cameras on the market. Zhang et al. [38] propose an implementation of a CNN accelerator using a cluster of FPGAs to achieve high performance and energy efficiency. Nguyen et al. [39] also use FPGAs to accelerate in particular the performance of the YOLO network to reduce power consumption relative to a GPU implementation. In most cases, the idea is to implement a processing element that can run convolutions efficiently, on which the different layers are sequentially mapped, storing the intermediate result in main or local memory. For instance, Silva et al. develop a highly configurable convolution block on an FPGA to accelerate object detection in autonomous driving applications [40]. Yan et al. also develop an accelerator on FPGA, optimizing the design using resource multiplexing and parallel processing, limiting the implementation to 3×3 kernels, to avoid issues with reconfiguration [41]. Sui et al. combine a low-bit power-of-two quantization method with an FPGA implementation that results in a processing element that uses shift operations only, avoiding the area and latency overhead of the multipliers [42]. Our approach follows a different idea, inspired by the need to compute the output as the stream of pixels is acquired in real time. In particular, rather than having a generic processing element where all layers are sequentialized, each layer is separately given resources according to the rate at which it computes, so that they all execute simultaneously. The layers are then interfaced with the required buffering to save just the amount of features required for a pipelined implementation. This is necessary to achieve the desired performance, since the network must execute at the speed of the input, which delivers 4 G pixels per second.

## 2. CNN and Quantization Background

Convolutional neural networks (CNNs) are a type of artificial neural network in which the connectivity pattern between neurons is inspired by the organization of the animal visual cortex [43,44]. In recent years, CNNs have proved to be a powerful and flexible tool to analyze images in many different applications. This involves the use of high-end hardware, with support for GPU computing or in the cloud, with the related higher cost, additional system complexity, and drawbacks in terms of on-premise deployment and installation. While the use of CNN for vision problems is not new [45], our idea is to design network architectures specifically intended to be easily implementable on limited hardware, in order to allow local processing (close to the image sensor) in real-time, removing the necessity to process the entire images externally. To this end, CNN quantization is often chosen in such scenarios to reduce execution time and system complexity [46].

Some of these methods propose to carry out the network computations using only integer arithmetic, taking advantage of its simplicity with respect to the floating point counterpart. A well-established approach was introduced in [16] and implemented in the modern TensorFlow Lite (TFL) framework. The idea is to provide an affine mapping of integers *q* to real numbers *r* in the form r=S(q−Z), where the *scale S* and *zero-point Z* are quantization parameters to be defined. Such parameters can be obtained in two ways:

1. By performing a *post-training* quantization, consisting of training the network with the usual floating point arithmetic, and then computing the parameters to minimize the quantization error of the resulting weights;
2. With a *quantization-aware* approach in which the forward pass of the training simulates the quantization so that the loss of precision can be compensated with potentially better weights. Note that, to overcome the vanishing gradient due to the integer arithmetic, the backpropagation is performed as usual and the weights and biases are still stored in floating-point for further processing.

The *quantization-aware* training often leads to better results because the loss function (but not its gradient) is computed considering the effect of quantization. Mathematically, the quantization function *q* is implemented as:

(1) $$\begin{array}{ccc} q(r,a,b,n) & = & \left\lfloor \frac{c(r,a,b)}{s(a,b,n)}\right\rfloor \;s\left(a,b,n\right)+a \end{array}$$

(2) $$\begin{array}{ccc} s(a,b,n) & = & \frac{b-a}{n-1} \end{array}$$

(3) $$\begin{array}{ccc} c(r,a,b) & = & \min(\max(r,a),b) \end{array}$$

where *a* and *b* are the extrema of the quantization range, *n* is the number of quantization levels, and *r* is the real number to quantize [16]. Usually, *n* is fixed depending on the amount of quantization required, whereas *a* and *b* are estimated on a per-layer basis according to the weight values observed during the training. Our approach loosely follows this general idea, but optimizes the quantization range during training together with the model variables.

The implementation of the inference phase in real applications also presents several challenges, especially when the data rate is large and information must be processed in real time. In these cases, it is difficult to resort to a cloud solution, because of the high latency involved with moving potentially large amount of information across the network [47,48]. Computation is therefore executed on edge devices, which must stand close to the data source (e.g., a video camera, a Lidar) but are typically constrained by the amount of resources and power available [49,50]. Quantization and pruning, as explained above, are therefore essential for an efficient implementation. At the same time, the high throughput required by certain applications is very hard to achieve with general purpose hardware, such as CPUs and GPUs, because of limitations in both memory and computation bandwidth, which appeal more to the average case [51]. While several integrated custom accelerators exist, they cannot be easily reconfigured to compute with arbitrary precision, reducing the hardware utilization and therefore performance. In this backdrop, FPGAs provide a customizable trade-off between speed, reconfigurability, and power consumption. The advantages of this kind of implementation have been reported in several studies. For instance, Wu et al. [52] conduct a detailed analysis of various optimization strategies, as they apply to both FPGAs and other custom solutions, highlighting in particular the flexibility of the FPGA reconfiguration capabilities. In the rest of the paper, we will analyze in detail the performance that can be achieved after quantization, and illustrate how a FPGA implementation can take advantage of the computation resources to achieve high throughput at low power consumption.

## 3. Arbitrary-Precision Quantized Layers

The main advantage of using FPGA hardware is the complete freedom in designing network components that can perform arithmetic operations with an arbitrary number of bits. On the other hand, classical methods do not comprise fully-adjustable quantized layers, so the potential advantage of using tailored hardware is somehow lost in the implementation and training steps.

The proposed approach involves the formulation of three custom layers, namely *quantized convolution*, *quantized fully connected*, and *requantizer*. The design of such layers is targeted at having the final network work with two’s complement addition and multiplication at *b* bits, with saturation arithmetic. This behavior is obtained by clipping (instead of wrapping) the overflowing values at the interval bounds $-2^{\left(b-1\right)}-1$ and $2^{\left(b-1\right)}-1$. Such operations can be efficiently implemented on the FPGA, but are difficult to obtain on standard CPUs, which are not designed for integer operations with a number of bits that is not a power of two (e.g., 3, 4, 5, 6 bits).

During the training phase, the custom layers work with floating point arithmetic, with operators used to simulate the quantization and saturation in the forward pass of the network. The effect is that, under the hood, operations are performed with floating point numbers, but the output produced by the network is exactly the same as what would be obtained with integer arithmetic. This allows us to execute the operations on modern GPUs that give maximum performance while working with 32-bit floats. However, when computing the gradient, we let the model behave as a standard non-quantized network by skipping the quantization operation. This avoids the vanishing gradient problem and allows a smoother gradient descent toward the optimum.

When the network is trained, each custom layer (with the associated quantized weights) can be synthesized to the FPGA to implement the true quantized operations in hardware at the inference time. The following sections describe the design of such novel components, together with our training-aware quantization procedure. Then, we describe how each layer can be realized in its hardware counterpart, together with the stream architecture used to maximize the throughput when using the network on continuous discrete-time signals.

### 3.1. 1D and 2D Convolution

The proposed technique involves standard 1D and 2D convolution operations. Each kernel $K_{i}$ of a convolutional layer *i* consists of a small window of weights, which slide on and are multiplied by the input tensor *X* to produce the output. For a 1D kernel with *m* weights, the convolution operation that produces the element of the output *Y* at coordinate *j* can be defined as

(4) $$Y\left(j\right)=\sum_{h=-m/2}^{h=m/2}X\left(j+h\right)\cdot K_{i}\left(h\right)$$

where m/2 is intended as integer division, and we index the weights of $K_{i}$ so that the middle is at index 0. When extended to all pixels, we denote the convolution as

(5) $$Y=X⋆K_{i}.$$

The output *Y* of the convolution is larger when the input *X* matches the weights of the kernel around the *j*-the pixel. A 2D operation is computed similarly, except that the kernel has two dimensions and slides vertically as well as horizontally. By combining several kernels and organizing them in layers, the network learns and extracts those features required for correct classification.

To implement the convolution operation efficiently, the weights of a kernel $K_{i}$ are quantized on the fly using $b_{i}$ bits during inference. To do so, the weights of the kernel are constrained to belong to the following set of integers:

(6) $$Q_{i}=\left\{m\in Z\;\;s.t.\;\;||m|\right|\leq M_{i}\}$$

where $M_{i}=2^{\left(b_{i}-1\right)}-1$ is the maximum absolute value in the defined interval, which depends on the quantization bits $b_{i}$. Note that (i) the number of bits $b_{i}$ is arbitrary and can be independently set for each layer and also modified during the training procedure, and (ii) the weights are symmetric with respect to the zero so that the convolution result is not altered in the null points.

The kernel constraint is enforced by a clipping function C applied directly to the values of weights of $K_{i}$, which can be tuned depending on the training requirements (see Section 3.4) and ensures saturation. This function is automatically applied during training such that the weights are projected to $Q_{i}$ after the gradient update. Since the gradient optimization is performed in the real domain (i.e., with 32-bit floats), the values in $K_{i}$ need to be rounded to match the integer requirement of the quantized convolution. Specifically, we define the rounding operation as:

(7) $$R\left(x\right)=x+\hat{I}\left(\left\lfloor x+0.5\right\rfloor -x\right)$$

where $\hat{I}\left(\cdot \right)$ is the identity function when doing inference and zero when computing the gradient (i.e., when doing back-propagation). In other words, the rounding operation is performed only during inference, but not during back-propagation: this allows us to keep the exact gradient for propagation and avoid instability as described in [32]. Note that our convolutional layer implementation has no bias because that operation is performed by the subsequent requantizer layer.

Mathematically, the quantized convolution is described as follows:

(8) $$Y_{i}=X⋆R\left(C\left(K_{i}\right)\right)$$

where ⋆ denotes the convolution operator, *X* is the input tensor and $Y_{i}$ is the *i*-th output. Such an operation guarantees that the output is an integer, but not necessarily in the set $Q_{i}$. When doing convolutions, the number of bits used for accumulating the products must be sufficient to hold the largest possible value that can be obtained in output. This is generally not a problem when implementing on an FPGA because most of the logic gates are spent to implement the multipliers and not for the registers used to hold the result. Note also that the input *X* can be quantized with a number of bits different from $b_{i}$. For example, in the first network layer we can have the input data quantized at 8 bits and each kernel quantized at 3. In this situation, we would synthesize in hardware a set of multipliers working with operands at 8 and 3 bits, respectively, storing the output to an 11-bit accumulator.

Our convolutions are linear with no bias since the non-linear activation part is realized by the *requantizer* that has to be present after each convolutional layer.

### 3.2. Fully-Connected

The quantized fully connected layer implements the classical linear combination between the input and a set of weights. As we did for the convolutional layer, we associate the *i*-th dense layer to a quantization level equal to $b_{i}$ bits. In practice, the dense layer is composed of a weight tensor $W_{i}$ and a bias $B_{i}$, both limited to have values from the set $Q_{i}$ as defined in Equation (6). Similarly to convolutions, it is mathematically described as:

(9) $$Y_{i}=R\left(C\left(W_{i}\right)\right)\cdot X+R\left(C\left(B_{i}\right)\right).$$

As before, *X* is the input tensor for the *i*-th layer and $Y_{i}$ is the output value. Also in this case, the output $Y_{i}$ can exceed the input quantized range. If the fully connected layer is not the last one, it should therefore be followed by a requantizer.

### 3.3. Requantizer

This unit serves as a trainable quantization layer, matching the constraints between subsequent convolutions (or between convolutions and fully-connected layers), as well as a nonlinear activation function. Each requantizer layer contains two trainable float scalar parameters α and β that are, respectively, a scaling factor and a bias to be applied to input data. The output of such layers is then computed as follows:

(10) Y=C(R(αX)+R(β))

where C is the clipping function, R is the standard rounding performed only during inference as defined in Equation (7), *X* is the layer input and *Y* is the layer output. Since the parameters α and β are optimized during the training process, this layer is able to dynamically adjust the range of output values by scaling and translating the input values, while introducing a non-linear function acting as a usual activation.

Note that the parameter β always behaves as an integer at inference time, but α will not because the rounding happens after the product with the input data. This is important to allow the values produced by the previous convolution to be scaled back to the required quantized interval. This is the only non-integer operation that we allow in the architecture. When implemented in the real FPGA, this floating point multiplication is approximated by an integer multiplication followed by a power-of-two division (i.e., a right shift). Experimentally, we observed no significant impact on the final accuracy when applying this approximation.

### 3.4. Quantization-Aware Training

Even if the gradient computation is not affected by quantization, the severe clipping that occurs if a small number of bits is used leads to an unstable training, especially at the beginning. To solve this problem, we propose a three-step process providing a slow transition from a hybrid partially-quantized to the final fully-quantized implementation. Each step lasts a variable number of epochs until the reduction in the loss function value between two subsequent epochs is less than a specified threshold.

#### 3.4.1. Step 1

In the first step, the network starts from random weights uniformly distributed in their respective quantization intervals $Q_{i}$. The clipping function is set to:

(11) $$C\left(x\right)=M_{i}\tanh\left(\frac{x}{M_{i}}\right)$$

combining the typical non-linear behavior of the tanh for activations and approximating the saturation arithmetic for values outside the quantization range since (11) has two horizontal asymptotes at $\pm M_{i}$. This function does not simulate exactly the saturation arithmetic, but its derivative is greater than zero throughout the whole domain. If a fully-connected layer is placed at the end of the network, as typically happens with classifiers, a classical non-quantized version is used here.

#### 3.4.2. Step 2

The clipping function in the kernel constraint for convolutional and requantizer layers is set to:

(12) $$C\left(x\right)=\max\{\min\left\{x,M_{i}\right\},-M_{i}\}$$

This way, the saturation arithmetic is properly simulated using floating point operations, but the gradient is zero outside the range $\left[-M_{i}\ldots M_{i}\right]$. However, after step 1 the network is close to the optimum and changing the clipping function has no catastrophic effect on the training as it would have if used at the beginning.

#### 3.4.3. Step 3

In the final step, the last fully connected layer (if present) is replaced with its quantized version described in Section 3.2. Since the quantization architecture can deal with an arbitrary number of bits, it is possible to slowly decrease the range of values available at each layer throughout the training process. For example, one can start with the first layer quantized at eight bits and then slowly reduce it to four bits throughout the epochs. In general, it is better to reserve more bits to the last fully-connected layer because it is critical for the final classifier performance.

## 4. FPGA Implementation

Our target applications require that the input be processed in real time, for a continuous stream of data with no gaps. In other words, the entire network must run at the pixel rate. For this reason, a traditional acceleration architecture, where processing elements (PE) are time-shared to sequentially compute the various layers, saving the temporary intermediate results, is unfeasible, at least for the foreseen data rates. Instead, we must employ a *streaming* architecture, where all layers operate in parallel, and data flows from layer to layer with only a minimum amount of storage. For this to work, weights are stored internally in dedicated buffers, as described below. The required performance is achieved by the use of pipelining.

In the rest of this section, we discuss the details of the implementation of the different phases of the layer execution. In particular, we describe how kernels are computed and how the hardware can be shared by different kernels if the rate of the input is lower than the clock speed. We then show how to introduce the max-pooling operator, and how to reformat the results of one layer to form the input of the following layer, using what we call an *aggregator*. When the input rate to a layer is particularly slow, an additional technique of kernel decomposition could be applied to save even more hardware.

### 4.1. Convolutional and Dense Layer

Each layer of the network operates on data coming from the previous layer (or from the input in the case of the first layer), organized as a one-dimensional stream of pixels, over a number of channels. Let *D* be the number of input channels. We denote the value of channel k∈[0,D] of pixel *i* as $p_{i}^{k}$. The convolutional operator mixes the values of all the channels of a window of pixels $\left(p_{0},\ldots ,p_{W}\right)$ of size *W* according to a weighted sum. If we denote by $w_{i}^{k}$ the *weight* applied to the value $p_{i}^{k}$, then the operator computes

$$c=\sum_{i=0}^{W-1}\;\sum_{k=0}^{D-1}w_{i}^{k}\cdot p_{i}^{k}+b$$

where *b*, when present, is the *bias* of the operator.

During operation, we receive *one* pixel per clock cycle (including all its channels). We could follow two approaches:

- We buffer the input until we have a window of pixels of size equal to the width of the kernel. At that point, the whole computation can be executed. When a new pixel is received, the window shifts by one place, to retain the correct values to be applied to the operator.
- We execute the products and the additions as soon as the corresponding data is available. Thus, instead of computing the two sums in one step, we divide the computation for each pixel over several clock cycles, and buffer the result of each *partial sum* to be used during the next cycle. For each cycle, we therefore compute only the inner sum. In this case, the bias is added only at the end of the computation.

The first approach is simpler to implement; however, it requires that a shifting window of pixels (and all its channels) also be stored between one layer and the next. With the second approach, on the other hand, we only need to store the result of the inner sum, instead of all the channels, therefore reducing the amount of required registers. We therefore follow this second strategy.

**Figure 1:**
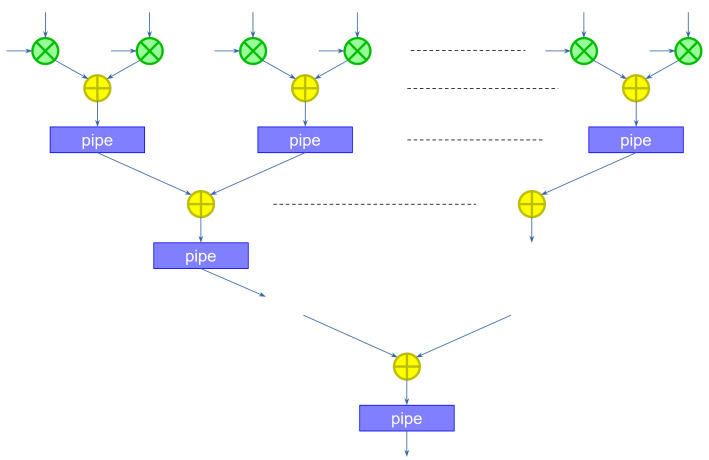
Kernel partial sum computation module. The multipliers are followed by a pipelined adder tree to compute a partial sum.

Figure 1 shows the hardware implementation of a kernel partial sum computation module.

Because the pixel is present at the input during a single clock cycle, the module multiplies every channel with its corresponding weight in parallel. The rest of the module implements the addition using an adder tree. The adders are separated by pipeline registers, to achieve the desired clock frequency. The result is available at the output after a delay of

$$\mathrm{delay}=\lceil \log_{2}\left(D\right)\rceil$$

clock cycles. The ceiling operator is required to account for a possible number of channels that is not a power of two.

While the input activations and the weights are specified to be of a certain size (number of bits), the size of the intermediate operators must be adjusted along the computation tree. One could try to retain the original size of the input at every step, by truncating (or approximating) the results of the operator. This would introduce too much error and significantly degrade the classification performance of the network. The alternative is to extend the size of the operators so that no information is lost. We accomplish this by keeping track of the highest and lowest possible values that the input (pixel and weights) can take, and by propagating that information along the computation graph.

**Figure 2:**
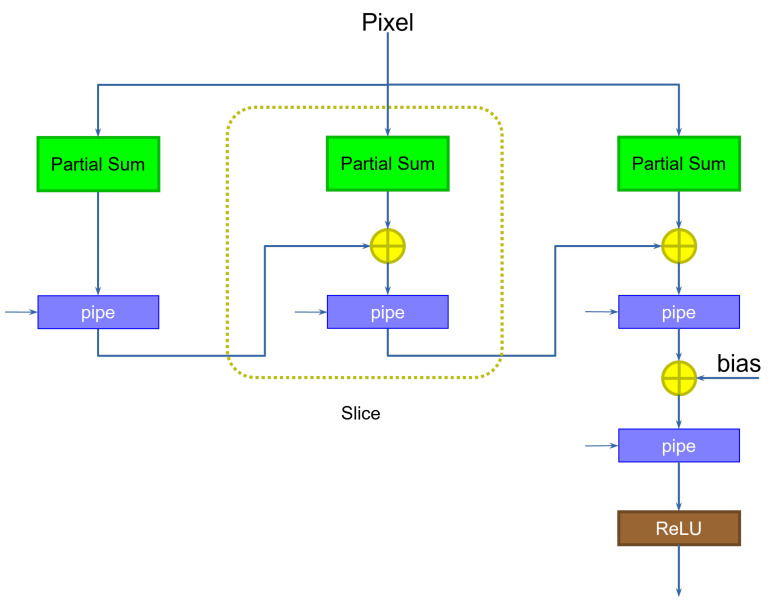
Sliced kernel computation. Different slices are connected in sequence to sequentially compute the full kernel.

When the first pixel is received, the network computes the first partial sum. When the second pixel is received, the same network *could* compute the second partial sum, accumulating the previous result. However, because the convolutional operator must slide through the entire image, a second instance of the kernel should start computing the first partial sum on the second pixel (assuming the convolution has a stride of 1). To keep up with the input speed, we need to compute these two partial sums in parallel. In practice, we need as many partial sum modules as the width of the kernel. The structure for a kernel of width 3 is shown in Figure 2.

During the first clock cycle, the first column (slice) on the left computes the first partial sum on the first pixel, saving the result in the pipeline register. During the second clock cycle, the first slice computes the first partial sum for the kernel translated one pixel to the right, on the second pixel, while the middle slice computes the second partial sum for the first kernel application, adding to the previous result. During the third clock cycle, the first slice computes the first partial sum of the next translated kernel, the second slice the second partial sum for the second translated kernel, and the third slice the final partial sum for the first kernel application, accumulating the result. The third slice also includes an additional step to add the bias. Note that the size in bits of the bias can be considerably larger than the size of the weights, since the size was increased as a result of the application of the accumulation. This reduces the approximation errors, and does not impact the hardware size too much, since the bias is added only once at the end of the computation. Finally, a ReLU component computes the non-linear output function.

If the layer contains several different kernels, the kernel computation must be replicated accordingly. The combination of the outputs of all the kernels forms the input of the following layer, producing all channels (one per kernel) of a pixel per clock cycle, thus preserving the original input rate.

*Dense* layers can easily be turned into convolutional layers of appropriate size, operating as the data become available from the previous layer. We therefore do not use a dedicated architecture, but instead make use of the same solution just described.

### 4.2. Rate Sharing

The max-pooling operator (see below) takes as input a number *W* of pixels, and outputs, for each channel, the highest value. Unlike the convolutional operator, which typically has a stride of 1, the max-pooling operator has a stride equal to its size. Therefore, the number of output pixels is reduced by the same amount. Consequently, when a max-pooling layer is applied, the output rate is also reduced by an amount equal to the size of the operator. If W=2, after one max-pooling, new features are generated every *two* clock cycles, after another max-pooling, every *four* clock cycles, and so on.

The lower pixel rate gives an opportunity for sharing resources among different kernels. Because the pipelined modules compute one result per clock cycle, if pixels come every two clock cycles, we can compute half of the kernels during the first, and the other half during the second clock cycle. This reduces the required amount of multipliers and adders by a factor of two. On the other hand, this raises two problems.

- The partial sum module must compute different weighted sums on alternate clock cycles. Consequently, weights cannot be provided as constants, but must be stored in buffers that are delivered to the multipliers at the right time.
- Because pixels come at a reduced rate, the partial result from one slice to the next must be delayed by a number of clock cycles equal to the rate at which data changes.

**Figure 3:**
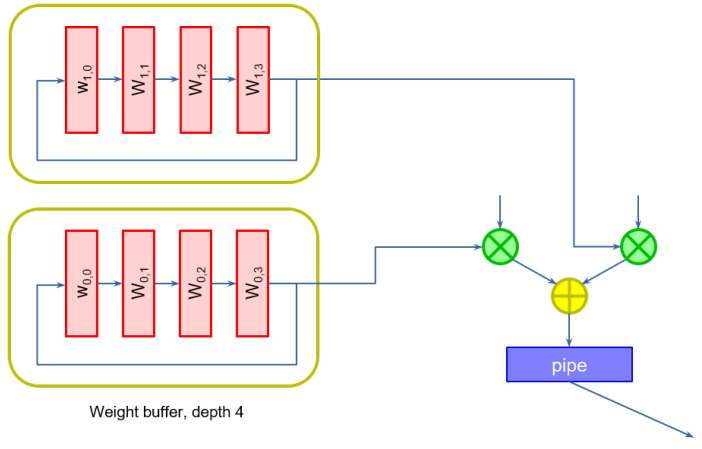
Weight buffers. A shift register is used to give the correct sequence of weights to the multipliers at every clock cycle.

We can easily accommodate these two requirements by modifying the structure of the slices. Weights are stored in a *weight buffer*. Because weights are continuously cycled to the multipliers, the selection can be conveniently operated by constructing a self-loaded shift register, instead of using a multiplexer, whose size would increase with the number of weights. Figure 3 shows an example of weight buffers of depth 4, used when data comes at a rate of one pixel every four clock cycles, for the first two multipliers.

The registers must be initialized with the desired values, either statically or dynamically, by adding an extra input to the first register of each buffer.

**Figure 4:**
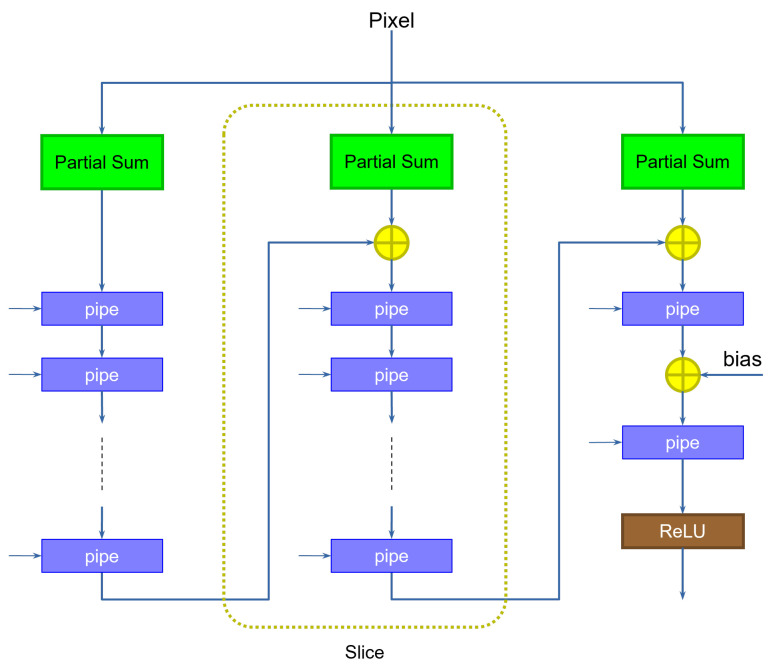
Partial sum accumulation with non-unit rate. Delay registers are used to store partial results as the kernels take turns computing in the partial sum module.

The second problem can be easily addressed by adding the appropriate number of pipeline registers at the output of each slice. Figure 4 shows an example.

The number of registers is exactly equal to the number of clock cycles per input. The original solution, shown previously on Figure 2, can be interpreted as the particular case in which data come at a rate of one pixel per clock cycle (hence only one pipeline register is required). Notice how the last stage does not need extra pipeline registers, as it does not feed any additional slice.

### 4.3. Requantizer

**Figure 5:**
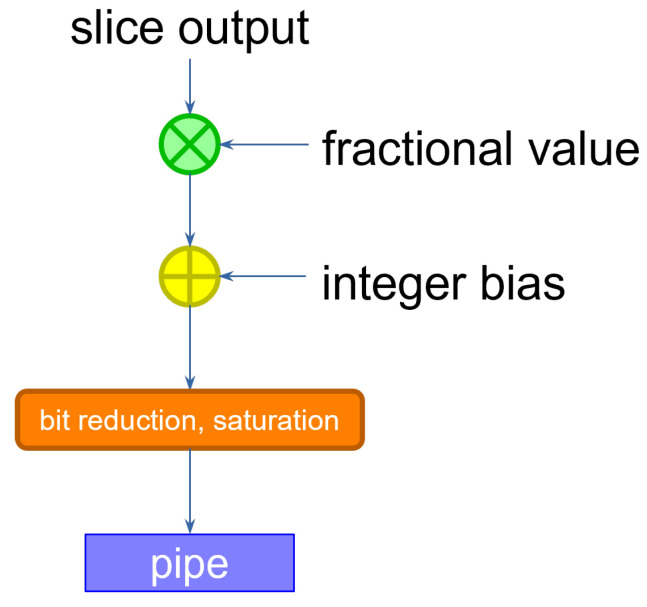
Value re-normalization between two layers. Integer arithmetic is used in this phase, by shifting the results by appropriate values.

The size of the data coming out of the slice is larger than that of the inputs and the weights. Because the input of the following layer is specified with a determined data size, the output activations must be re-normalized by discarding the extra bits. This can be accomplished by multiplying the result by a scaling factor. If the input activations are sized equally across the layers, we expect the scaling factor to be lower than 1. The operation can therefore be performed by multiplying the slice output by a purely fractional value, then adding a bias, and finally retaining the integer bits that correspond to the size accepted by the following layer, discarding the fractional result [16]. If the result were to exceed that size, then we simply saturate the value to the maximum representable quantity. The corresponding circuit is shown in Figure 5.

### 4.4. Max-Pooling Operator

The max-pooling layer compares the output of two (or more) adjacent pixels on the same channel, and outputs the largest value. As discussed previously, the operation typically uses a stride equal to its size, so that the output image is reduced. Here, we consider only the max-pooling operation of size 2.

**Figure 6:**
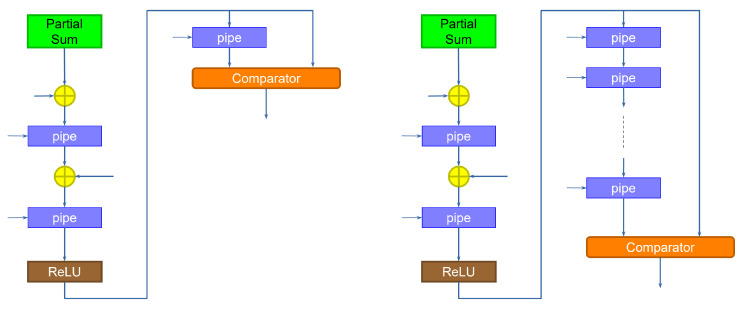
Implementation of the max-pooling operator. Outputs are compared to the previous result by a delay that depends on the rate at which the stage is operating.

Max-pooling can be implemented at the output of the last slice of computation. The structure is shown in Figure 6.

The left side of the figure shows the implementation for a layer working at rate 1 (one pixel per clock cycle). The idea is to compare the output of the stage with a delayed version (going through a pipeline register) of the output, which represents the value of the previous output pixel. The comparator then outputs the largest value. The implementation on the right is used for a layer that works at a slower rate. In this case, the slice takes several clock cycles to output a new pixel, while it computes the values of different channels (kernels). Therefore, a new pixel is output only after a number of clock cycles equal to the rate of the input. The operator must therefore delay the values, through a corresponding number of pipeline registers, so that the comparison takes place between the channel of one pixel and the same channel of the next pixel.

Notice how in both cases the operator computes a new result *every* clock cycle. This is contrary to the assumption we made on the stride. For instance, in the first case (left of the figure), the comparator should output a new value only every other clock cycle. There are two possible solutions to this. The first is to have the following stage be aware of the behavior of max-pooling, and simply ignore every other pixel. This solution makes the construction of the network difficult, as each layer would depend on the previous. To promote a more modular design, we instead employ a stage dedicated to *aggregating* the output values. This stage serves two purposes:

- A signal is used to load a new value only when the input is valid. Proper sequencing of this signal can be used to discard the spurious computations of the max-pooling operator.
- A set of slices is again used to aggregate the pixel values as they are generated by a layer working at a rate lower than one pixel per clock cycle. The pixels are then forwarded to the next layer only when the data corresponding to all the channels is available.

By doing so, a stage is not aware of the specific sequencing operations applied in the previous stage, but instead sees all the pixel channels valid at the same time for the correct number of clock cycles that corresponds to its rate.

**Figure 7:**
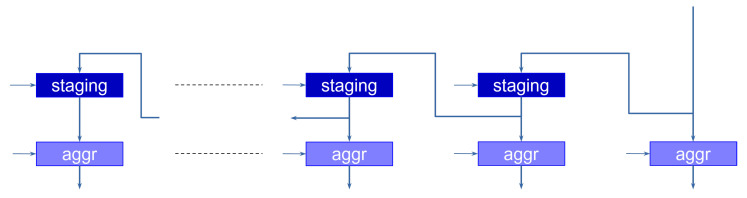
Aggregation module. Outputs are staged in temporary registers until all values are ready to be transferred to the next layer.

Figure 7 shows the implementation of this stage.

The operation goes through two phases. During the first phase, the data coming from the layer (the output of the last slice or the max-pooling operator) is shifted along the *staging* registers, which are enabled every time a new value is available at the input. This allows the aggregator to discard the invalid outputs of the max-pooling operator. The second phase takes place when the last values (e.g., the last channels) are available at the input. In this case, the aggregation registers are enabled, loading the data in parallel, and presenting it at the same time to the next layer.

The operation of the max-pooling and aggregation stages are controlled by the periodic load enabling signals of the registers, which are generated by an appropriate sequencing module.

### 4.5. Kernel Decomposition

**Figure 8:**
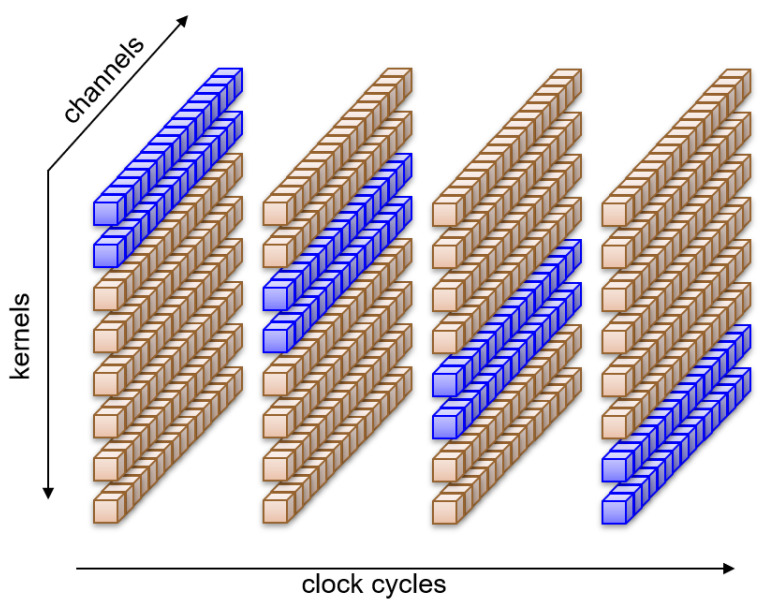
Rate sharing: different kernels are computed along all channels in different clock cycles.

With the rate-sharing technique outlined above, we distribute the computation of whole kernels along the available clock cycles. The approach can be shown graphically in Figure 8, where eight kernels alternate in pairs to compute all sixteen channels at the same time.

**Figure 9:**
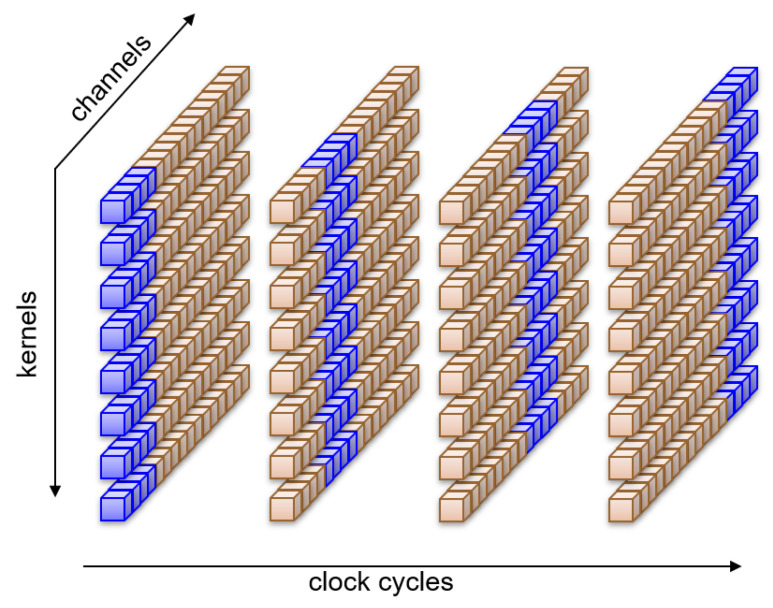
Kernel decomposition: different channels are computed for all kernels in different clock cycles.

After several max-pooling operations, the rate at which pixels reach a layer may be lower than the number of kernels present in the layer. Then, even if we run only one kernel per clock cycle, there will be clock cycles in which the operators will remain idle, having no further computations to perform. The extra time available could be used to improve efficiency by diluting the computation of a kernel over several clock cycles. This will expose even more opportunities for sharing resources. In fact, one could decompose the kernel computation instead of using the rate sharing technique described above even when the rate at which pixels reach a layer is higher than the number of kernels. This paradigm is shown in Figure 9, where all eight kernels compute at the same time on only a portion of the sixteen channels for each clock cycle.

In this case, we must accumulate results from different channels in time. The advantage is that all kernels terminate their computation at the same time, and therefore we need fewer pipeline registers (in many cases only one) after the slice. At the same time, we have correspondingly more kernels, which require a separate implementation, and a separate pipeline register. Hence, the final number of pipeline registers (and multipliers) is the same. Kernel decomposition, however, also requires an accumulator, which is not present in rate sharing.

**Figure 10:**
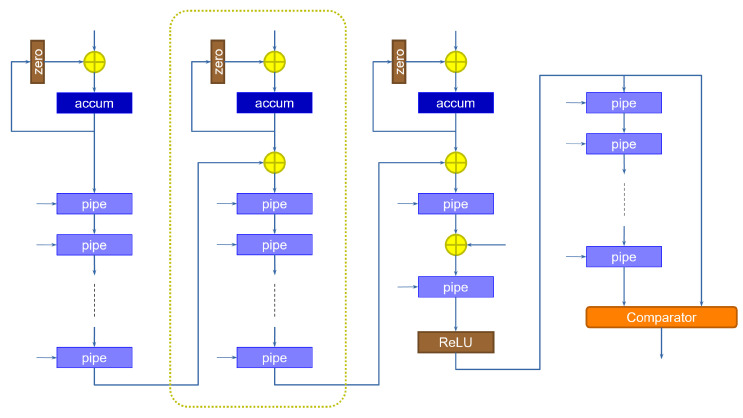
Accumulation module. An accumulator is used to store the partial result within the same slice.

The architecture of the accumulation portion of the computation must be extended, because results must now be accumulated within the same slice. One possible implementation of this solution is shown in Figure 10, which extends the previous slice architecture with an accumulation function.

The data coming from the adder tree are stored in the accumulator, whose value is then fed back to itself to complete the summation. The “zero” block is used to zero-out the output of the accumulator, when we want to initialize it to a new value coming from the slice, every time a new pixel is received. This is essential, as we do not have a spare clock cycle to be used to bring the accumulator back to zero. The accumulation is performed as many times as the number of cycles required to cover all the channels. At that point, the value is transferred onto the pipeline registers by enabling them to load. As discussed above, this solution requires fewer pipeline registers than the rate sharing technique, as the pipeline registers are activated only at the end of the accumulation instead of every clock cycle. On the other hand, because we run all kernels concurrently, more blocks of this kind must be instantiated, with the corresponding registers and max-pooling elements.

## 5. Experimental Results and Discussion

Our approach was tested on the case study of peak detection for one-dimensional signals. This task is fundamental in many vision-based industrial setups, for example as a first stage in 3D laser-based triangulation systems. In such approaches, a narrow stripe of light generated from a laser is projected onto a 3D surface, resulting in an illuminated curve when captured by a calibrated camera. The camera is placed so that the captured laser line is almost orthogonal to image y-axis: in this way we can estimate the 3D shape of the original surface by searching for brighter peaks along each camera row. This process is highly parallelizable and can take place near the camera, for example using an FPGA. Typical algorithms use single or multiple thresholds to perform peak detection; however, this shows several drawbacks and cannot always ensure good performance. First, the background is not uniformly dark as it can contain regions that are brighter than the laser stripes. Second, the reflected signal depends on the surface albedo: it can be very weak on dark surfaces, and too bright if the underlying object is white. Defining a unique threshold for the whole scene is very problematic in this case. Third, there might be reflections around the main laser line in the case of highly reflective surfaces such as metals. For the broad variety of visual conditions to deal with, it makes sense to use a learning based model trained to discriminate real peaks from noise: we designed such model as a convolutional neural network, sufficiently small and quantized to be implemented onboard the camera on a custom FPGA.

### 5.1. Network Architecture

To limit the complexity and keep it independent from the size of the input, we designed our network as a standard feed-forward classifier. The input is composed by batches of 16 pixels, selected by moving a sliding window over the acquired scan-line. Each batch is classified into three possible classes, according to whether there is no peak in the batch (encoded as class 0), the peak is in the first half of the batch (class 1), or the peak is the last half of the batch (class −1). At inference time, the whole signal is processed to produce a sequence of classes, then the crossings between class −1 and 1 are marked as peaks.

We started with a model composed by two convolutional layers C1,C2 interleaved with max-pooling and followed by a final fully-connected layer. C1 and C2 were composed by 32 kernels each of size 3 with ReLU activations. The fully-connected layer produced an output tensor of size 3 (one-hot encoding of the 3 classes) through softmax activation. With a simple pruning procedure, similar to [53], we reduced the size of the network, removing unnecessary kernels. The algorithm ranks the filters based on their output (after the activation) when predicting a random sample of the training set. Kernels producing zero (or close to zero) output values receive a low ranking score since they contribute less in defining the output class. The filter with lower rank is dropped and the network is fine tuned to compensate the absence of the removed kernel. The procedure was repeated until we observed an accuracy drop higher than 3% from the initial one. After pruning, layers C1 and C2 were reduced to just 6 and 3 kernels, respectively. At this point, we replaced convolutional and fully-connected layers with their quantized counterparts, and the network is trained from scratch with the 3-step procedure discussed before.

### 5.2. CNN Quantization

**Figure 11:**
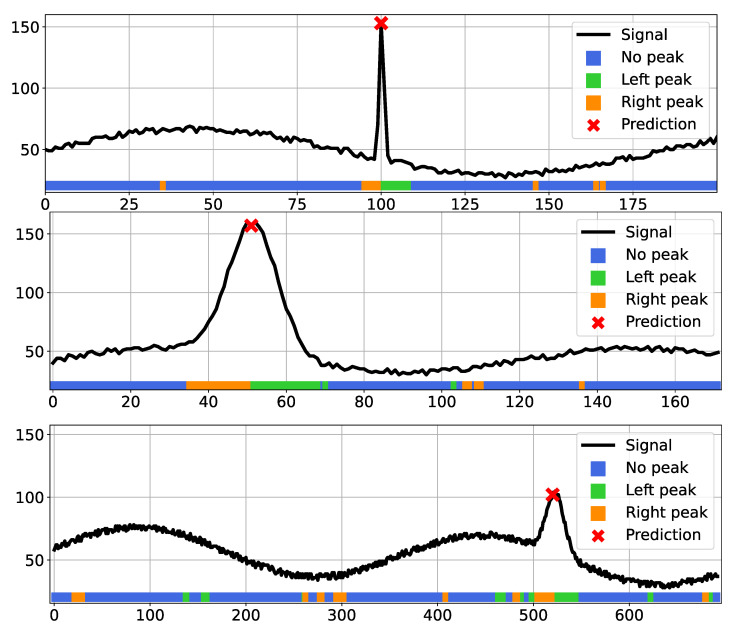
Some examples of one-dimensional signals (in black) from our synthetic dataset generated to simulate camera scanlines for the peak detection network. The displayed samples are taken from the test set and each colored stripe denotes the predicted classes for each signal portion, namely: no peak, left peak and right peak. The red cross denotes the peak location computed from the given prediction.

**Figure 12:**
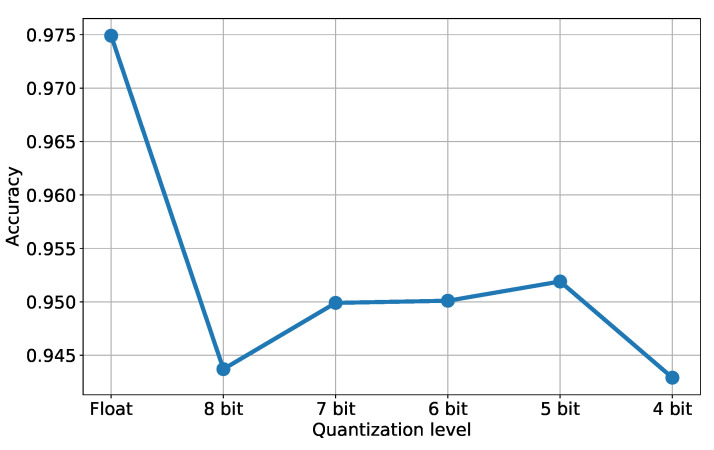
Accuracy values for the proposed method and different quantization levels. The leftmost value “Float” indicates the original network with no quantization, while other values show the accuracy obtained decreasing the quantization from 8 down to 4 bits.

To assess the proposed framework, we first tested the performance of the quantization method itself and its impact on the network accuracy when used as a simple classifier. We compared with other state-of-the-art quantization methods implemented by the TensorFlow Lite (TFL) library as described in [16,54]. We applied both post-training quantization and quantization-aware solutions with different settings, as described in the documentation. In this initial experiment, we employed fully synthetic data for both training and testing: we generated a set of 100 K 1D signals of 1024 pixels, each with one or more peaks to be detected. The signals exhibit a variable random-generated noise and some other patterns to simulate different acquisition conditions, and the ground truth peaks have different width and height. Three representative scanline portions from such datasets are shown in Figure 11 in black. We divided the train and test sets with a 70/30 ratio and trained all models until convergence with the Adam optimizer and an adaptive learning rate starting at $10^{-3}$. Table 1 shows the test set accuracy after applying different quantization techniques to the original model (in float32, displayed in the first row). We tested TFL quantization tools in three variants: “dynamic range” converts floating point weights into integers but performs inference with floating point values, while “integer-only UINT8” and “integer-only INT8” perform integer-only operations. Note that the integer-only conversion needs a representative dataset as an input to calibrate the range for the network tensors. The table also shows our eight- and four-bit fully-quantized networks (rows 2 and 3) and the same two architectures with a binarized dense layer. The accuracy values highlight the potential of our approach with respect to standard approaches without requiring a representative set of data. In general, we observed a decay in accuracy when introducing binarized layers, so we continued with the four-bit version of our architecture. We also tested our approach with different quantization levels, namely eight, seven, six, five, four, and four bits. The associated accuracy values are plotted in Figure 12, where the first value on the left corresponds to the original model (i.e., the floating point version). As expected, we can notice a significant decrease in accuracy after the first quantization, when moving from Float to eight bits of accuracy. The other results (from eight down to four bits) basically show a stable behavior around an accuracy value of 0.95. The colored stripes below each signal in Figure 11 show the predicted class for each input pixel as a different color when running prediction with our four-bit quantized network. The majority of pixels are correctly labeled as “no peak” (blue), while in the proximity of the actual signal peaks we can notice a clear pattern composed by a sequence of “right peak” (orange) class pixels followed by “left peak” (green) pixels. The combination of such a sequence of classes makes the peak detection effective and allows us to compute the position of the actual peak, despite some misclassified pixels (seen as isolated right or left peaks) that are ignored by the system.

The second experiment we performed is meant to verify the quality of the detected peaks: we compared our learning-based peak detection with a set of classical algorithms usually employed for this kind of task. We implemented three methods, namely *center of gravity*, *max threshold* and *FindPeaks*. Center of gravity uses a single threshold and identifies the peak as the center of gravity of all the values above the threshold, while max threshold works with two thresholds and computes the peak as the average of the (possible) intersections with the signal. Such algorithms are widely used in standard peak-detection approaches and also implemented directly on hardware, for instance as shown in [55,56]. FindPeaks performs signal shape analysis, identifying regions where the shape matches a bell curve: it uses two thresholds, one for signal intensity and one for peak width to extract one or more peaks from a given scanline. Since the first two methods assume a single peak to be detected, we restricted our analysis to signals with a single peak, keeping in mind that our architecture is able to recognize an arbitrary number of peaks. In our case, the peak location was computed by fitting a parabola through the points corresponding to the class change from “right peak” to “left peak”. Table 2 reports the comparison results in terms of peak detection accuracy and peak localization mean absolute error (MAE). The displayed accuracy denotes the number of correctly detected peaks over the total number of peaks generated in the dataset: we considered a peak as successfully identified if the detected peak is less than 10 pixels away from the true location. We compared three different versions of our architecture, which include the non-quantized original network (denoted as Float32), and the eight- and four-bit networks quantized with the proposed approach. Our CNN approach offers better detection performance in both standard and quantized versions, being consistently above 90% in terms of accuracy: in particular, the eight-bit quantized model achieves an accuracy that is comparable with the full model (respectively, 0.937 and 0.938), denoting how the quantization process is indeed effective in preserving the required performance. On the other hand, algorithmic peak detection algorithms report an accuracy always below 0.9. In terms of peak localization error, all the proposed learning-based techniques exhibit an average error always below 0.5 pixels. Such an error is quite small for the full and the eight-bit models (0.210 and 0.348), while for the other approaches it is significantly larger, even greater than one pixel.

**Figure 13:**
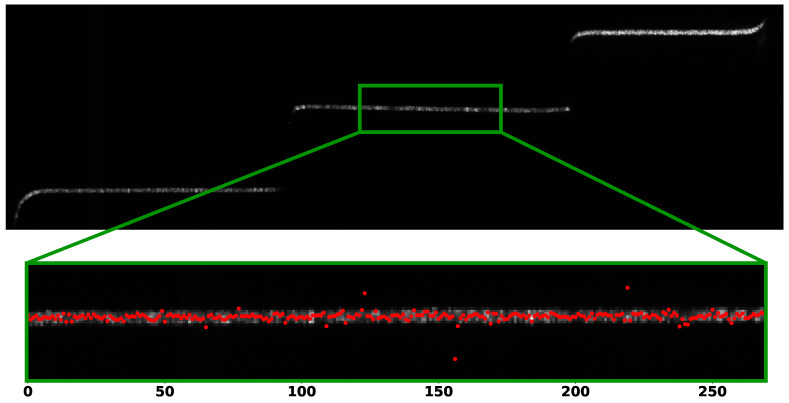
Example of a real acquired image with a laser line hitting a metallic object formed by three planar parts (**top**) and a line detail (**bottom**) with peaks detected by our 4-bit quantized network plotted as red points.

As a last experiment, we tested our approach on a real-world setup. We acquired a metallic object hit by a single laser line and processed the resulting images with our CNN and the other peak detection algorithms. We employed a grayscale camera with a resolution of 2048×1024 pixels and a field of view of 25 mm, mounted at 75 mm from a blue laser line with an angle of $30^{\circ }$. An example of the acquired scene is shown in Figure 13 (top): the captured object is not visible due to the dark environment, but three planar surfaces are hit by the laser line, resulting in three segments on the image plane. The object material (brush metal) makes the acquired signal very noisy, so that in practice the peak detection process for each camera column is quite challenging. Since the ground truth is not available, we exploited the fact that the projected laser line generates a straight line when intersecting the planar surface in the central region of the three segments. We thus selected some areas of interest in the scene (see the details in the green box in Figure 13), run the peak detection algorithm for each image column and fitted a line through all the detected points (plotted in red). Table 3 shows the Root Mean Square (RMS) and standard deviation of the distances of detected points from the fitted line as a measure of the peak detection quality. Peaks were detected with the previously discussed methods. Also in this case, the resulting values show a smaller RMS for our CNN-based approaches with respect to other classical methods, exhibiting a higher stability for peak detection in such challenging real-world scenarios.

### 5.3. FPGA Experiments

We have implemented the networks described above to evaluate the feasibility of the approach on embedded hardware. As described above, we take the wood industry as a reference, using as an example a CNN architecture for peak detection in a stream of pixels coming from a high-speed camera. The requirements are stringent: the network must be able to operate in real time, processing 16 separate streams in parallel while minimizing resource utilization, for a total of 4 G pixels per second. The required high data rate can be obtained by an appropriate use of parallelism and pipelining, leading to a clock frequency of 250 MHz. The target implementation platform is the Zynq UltraScale+ MPSoC ZU9EG FPGA hosted on a ZCU102 board from Xilinx. This is a rather large device, which we use to study the scalability of our approach. Many of the implementations will make use of only a portion of the available resources. Note that while this device contains a Quad-core Arm Cortex-A53 processor and a Dual-core Arm Cortex-R5F, we do not make use of these resources and limit our exploration to only the Programmable Logic (PL) part of the FPGA.

Given the highly regular structure of the network, we found it convenient to develop an automatic procedure, written in roughly 10,000 lines of C++ code, that generates the VHDL description from a specification of the layers akin to the level of TensorFlow. This allows us to quickly generate a new implementation, to explore the design space and rule out solutions that do not satisfy requirements in terms of area and performance. The networks presented in this paper are the final results of this exploration process. The code generator also keeps track of the range of all operands along the network, in particular in the adder trees, to correctly size the operators (i.e., the number of bits) and avoid potential overflow situations. All synthesis jobs are conducted with Vivado v2020.2 (64-bit) running on Ubuntu 20.04. To streamline the operations, we use non-project mode with a script running the default technology independent optimization, place and route, and physical optimization, generating the final bitstream. The network inputs and outputs come from internal BRAMs (Block RAMs), which are continuously fed from external RAM where the data from the camera are stored.

The pixels from the camera are organized into 16 separate scanlines, called *frames*, which are processed independently. To meet the timing requirements, the basic CNN architecture is replicated 16 times, once for each frame. On the other hand, because the networks process the data synchronously, the weight buffers can be shared and do not need to be duplicated. Table 4 shows detailed results for the reference CNN architectures that were evaluated in the previous section. We consider only fixed-point implementations quantized at eight and four bits, as the floating-point implementation is too large to fit even the target device. The table shows the number of layers, the number of trainable parameters, the quantization in number of bits, and the total number of multiplications needed. We evaluate the utilization in terms of Configurable Logic Blocks (CLBs), Look-Up Tables (LUTs), Registers (Regs) and dedicated DSP (Digital Signal Processor) logic. The number under the headings indicate the total available resources on the device. The table also shows the achieved performance in terms of clock frequency and the corresponding slack, as well as the total power consumption as estimated by Vivado using the default configuration.

For each quantization, we consider an implementation that uses exclusively the logic for computation, and one that replaces the logic with the available dedicated DSPs. We take advantage of the SIMD (Single Instruction Multiple Data) capabilities of the DSPs using the synthesis attributes, to share DSPs across several operators. With 142 trainable parameters per frame, we expect a total of 142×16=2272 multipliers. The actual number of multipliers is actually almost half that value, because of the sharing made possible after the first layer, where the data rate decreases. The implementation includes also roughly as many adders (of various sizes) for the adder trees, and some additional resources for the re-normalization. Overall, the architecture computes 4544 operations per pixel (multipliers + adders), for an ideal performance of $4544\times 250\times 10^{6}=1.14$ TOPS.

The results show that using 8-bit quantization, the 16-frame implementation uses a considerable portion of the FPGA (almost half) when not employing the DSPs, and achieves a clock frequency close to the target 250 MHz. The use of DSPs dramatically reduces the logic utilization; however, it incurs a penalty both in terms of cycle time and power consumption. Observe that the CLB utilization is higher than the LUT utilization. This is likely because the design is spread over the device (to access the DSPs), and part of the CLB goes unused. Other components that might be present on the device could make use of these spare resources. The four-bit implementation is significantly smaller, and could fit in much smaller and cheaper devices. The timing constraints are essentially satisfied, while power consumption is much lower, due to the smaller size of the operators. The last row of the table shows the same architecture when synthesized with a target 333 MHz clock frequency. In this case, the tool is able to achieve higher performance, achieving a clock frequency of 262 MHz, with a small penalty in terms of LUTs and power consumption (surprisingly, it can make better use of the CLBs). For this particular architecture, running at the nominal 250 MHz rate, with a power consumption of 2.05 W, we achieve a *sustained* efficiency of 1.14/2.05≈0.5 TOPS/W. This compares favorably to several dedicated custom accelerators [57]. In particular, Reuthers et al. compare a large number of different accelerator architectures, and show that, on average, they achieve a *peak* performance of roughly 1 TOPS/W (Tera Operations per Second per Watt) across different technologies [58]. For these accelerators, the actual efficiency will then depend on the achieved level of hardware utilization, and could be considerably lower.

Table 5 shows additional data on different architectures that we have synthesized for our exploration. These are *simplified* models that do not include re-normalization and memory interfacing, and are meant only to quickly explore the design space. We show results for larger networks made of twelve and five layers, with quantizations using eight, four and one bit. The one-bit quantization corresponds to an XNOR implementation with pop-count [32,33,34], and is included only for reference as its performance in terms of classification accuracy was not evaluated. Large unpruned 12-layer networks cannot be made to fit into the target device for the complete 16-frame system. In fact, even one frame for the eight-bit implementation almost fills the device. The pruned 12-layer version, with less than half the parameters, is much smaller and can be used for the 16-frame system. Reducing the network to five layers correspondingly reduces the area utilization. We notice how the binarized networks are far smaller than the corresponding quantized versions, and handily meet the timing requirements. This suggests that (i) the degree of pipelining could be considerably reduced for binarized networks, thus further reducing resource utilization, and (ii) more complex binarized networks could replace simpler architectures, if they achieve the desired accuracy [35]. We have not yet fully explored this opportunity, and reserve it for our future work.

Finally, we observe that as we replicate the hardware for the desired number of frames, the logic utilization grows more than anticipated. Table 6 shows detailed data for the five-layer four-bit implementation as the number of frames is increased from 1 to 16. In particular, we see that despite the weight sharing, the proportion of LUTs for weight buffers (last column) as reported by the tool does not decrease as much as expected. This may be due to the higher resources required to route the weight data to the frames, as well as the additional difficulty in meeting the timing requirements as the design becomes larger. Replicating the weight buffers to reduce routing and delay did not improve the results.

## 6. Conclusions

We proposed a simple yet effective way to train a quantization-aware neural network composed of layers that can operate with saturation arithmetic working at an arbitrary number of bits. The layers are trained on classical GPUs and then synthesized to FPGA hardware for real-time inference. A trainable *Requantizer* component acts both as a non-linear activation for neurons and a value rescaler to match the desired bit precision. This way, the training is not only *quantization-aware*, but also capable of estimating the optimal scaling coefficients to accommodate both the non-linear nature of the activations and the constraints imposed by the limited precision. We presented a case study of a learning-based peak detector for laser scanning demonstrating how the proposed approach can outperform non-learning-based approaches in terms of accuracy, resilience to noise and speed. In particular, the networks achieve an accuracy of 94% when quantized with eight- and four-bit integers, and show promising results with accuracy reaching 87% when using binarized networks, without the need of calibration data required by other methods, such as TensorFlow Lite. The RMS peak localization error is lower than 0.5 pixels, and lower than the error of three peak detection specific algorithms both for synthetic and real data. Our approach is therefore not only competitive with deterministic algorithms, but supports a high degree of reconfigurability by simply changing the value of the weights, providing that needed level of flexibility required by applications with changing requirements. Such layers can be implemented efficiently in a FPGA to run the inference directly onboard industrial cameras. The experiments on a FPGA show that the model can achieve the desired real-time high throughput of 4 gigapixels per second using moderate hardware resources (depending on the quantization level), with favorable results in terms of power efficiency, which is close to that achieved on average by integrated custom neural network accelerators. As future work, we plan to expand the methodology to different kinds of layers to implement more sophisticated models.

## Figures and Tables

**Table 1:** Accuracy comparison for different quantization techniques. The first line, “Float32”, denotes the original network model with no quantization. Our approach is applied in four versions: 8-bit, 4-bit, 8-bit binarized and 4-bit binarized; where binarized refers only to the last fully connected layer (see network architecture section for more details). We compared with different quantization approaches proposed by the TensorFlow Lite library as described in [16,54].

Model	Quantization	Accuracy
Float32 (no quantization)	-	0.9749
Our 8-bit	range [−127, 127]	0.9437
Our 4-bit	range [−7, 7]	0.9429
Our 8-bit + binarized	range [−127, 127]	0.7393
Our 4-bit + binarized	range [−7, 7]	0.8795
TFL post-training	Integer-only UINT8	0.9673
TFL post-training	Integer-only INT8	0.7635
TFL post-training	Dynamic range	0.9385
TFL quantization-aware	Integer-only UINT8	0.9320
TFL quantization-aware	Dynamic range	0.9317

**Table 2:** Comparison between our learning-based peak detection method and classical algorithmic approaches. Our model is reported in three versions: the original non-quantized model (Float32) and two different quantization settings. For each technique, we display the ability in detecting a peak in a scanline (peak accuracy) and the peak localization error with respect to the ground truth (as a mean absolute error, in pixels). All tests were performed on our peak test set, for a total of 30,000 samples.

Algorithm	Peak Accuracy	MAE (px)
CNN Float32	0.938	0.210±0.446
CNN 8-bit quantized	0.937	0.348±0.566
CNN 4-bit quantized	0.914	0.436±0.607
FindPeaks	0.828	0.934±0.574
Center of Gravity	0.898	1.061±0.596
Max Threshold	0.897	1.226±0.666

**Table 3:** Tests on real-world images: RMS values after line fitting for different peak detection algorithms, namely: our proposed CNN model in three variants and classical peak detection. The values in bold denote the solution with the lowest error.

Algorithm	Line Fit RMS (px)
CNN Float32	0.841±0.510
CNN 8-bit quantized	**0.563** ± **0.374**
CNN 4-bit quantized	0.653±0.445
FindPeaks	0.716±0.403
Center of Gravity	0.731±0.515
Max Threshold	0.708±0.493

**Table 4:** Resource utilization for reference network architectures.

Configuration			16 Frames						
**Layers**	**Param.**	**Bits**	**Mults**	**CLBs**	**LUTs**	**Regs**	**DSPs**	**Clock MHz**	**Power W**
				(34,260)	(274,080)	(548,160)	(2520)	(slack (ns))	
3	142	8	1248	16,038	79,561	27,437	0	239	3.89
				(46.81%)	(29.03%)	(5.01%)	(0%)	(−0.173)	
3 (dsp)	142	8	1248	2857	7392	7064	1904	178	4.05
				(8.34%)	(2.70%)	(1.29%)	(76%)	(−1.616)	
3	142	4	1248	6118	31,669	15,474	0	246	2.05
				(17.86%)	(11.55%)	(2.82%)	(0%)	(−0.070)	
3 (dsp)	142	4	1248	2093	4390	4603	1680	202	3.25
				(6.11%)	(1.60%)	(0.84%)	(67%)	(−0.954)	
3	142	4	1248	5989	33,146	15,474	0	262	2.52
				(17.48%)	(12.09%)	(2.82%)	(0%)	(0.183)	

**Table 5:** Scalability of resource utilization.

Configuration			1 Frame			
**Layers**	**Param.**	**Bits**	**Mults**	**CLBs**	**LUTs**	**Slack (ns)**
				(34,260)	(274,080)	(4 ns)
12	7622	8	2456	86.28%	61.37%	0.047 ns
12	7622	4	2456	18.19%	13.43%	0.523 ns
12	7622	1	2408	2.64%	1.65%	1.760 ns
12	3243	4	611	6.84%	5.00%	0.676 ns
12	3243	1	611	1.04%	0.62%	2.065 ns
5	383	4	233	1.53%	0.94%	1.768 ns
5	383	1	230	0.25%	0.14%	2.937 ns
**Configuration**			**16 Frames**			
**Layers**	**Param.**	**Bits**	**Mults**	**CLBs**	**LUTs**	**Slack (ns)**
				(34,260)	(274,080)	(4 ns)
12	7622	8	-	-	-	-
12	7622	4	-	-	-	-
12	7622	1	38480	34.75%	24.57%	0.973 ns
12	3243	4	9776	85.90%	61.35%	0.215 ns
12	3243	1	9776	11.53%	8.13%	1.294 ns
5	383	4	3728	21.19%	13.74%	0.866 ns
5	383	1	3488	3.09%	1.91%	1.685 ns

**Table 6:** Scalability of the peak detection network, 5-layer 4-bit implementation.

Frames	Slack	Ops	CLB	Tot	Weight Buf.
	(ns)		%	LUTs	LUTs (%)
1	1.768	233	1.53	2575 (1.00×)	493 (19.1%)
2	1.492	466	2.78	4803 (1.86×)	746 (15.5%)
4	1.339	932	5.55	9446 (3.67×)	1385 (14.7%)
8	1.284	1864	11.21	19,130 (7.43×)	2825 (14.8%)
16	0.866	3728	21.19	37,647 (14.6×)	5254 (14.0%)

## Data Availability

The data presented in this study are available on request from the corresponding author. The data are not publicly available due to the industrial and proprietary nature of the information.
